# Multimodal MRI reveals structural and functional alterations in isolated cervical dystonia: associations with motor severity and affective symptoms

**DOI:** 10.3389/fneur.2026.1771144

**Published:** 2026-02-23

**Authors:** Qingwei Guo, Yaru Luo, Naixuan Du, Jinyang Li, Zhiyuan Yang, Zhongyuan Xia, Jiongyue Yun, Jihua Liu

**Affiliations:** 1 Department of Radiology, First Teaching Hospital of Tianjin University of Traditional Chinese Medicine, Tianjin, China; 2 National Clinical Research Center for Chinese Medicine, Tianjin, China; 3 Department of Anesthesiology, Renmin Hospital of Wuhan University, Wuhan, Hubei, China; 4 Department of Tuina, First Teaching Hospital of Tianjin University of Traditional Chinese Medicine, Tianjin, China; 5 Department of Medical Equipment, Tianjin Medical University Baodi Hospital, Tianjin, China

**Keywords:** anxiety, gray matter volume, isolated cervical dystonia, multimodal neuroimaging, resting-state fMRI

## Abstract

**Introduction:**

Isolated cervical dystonia (ICD) is the most common focal dystonia, characterized by involuntary neck muscle contractions leading to abnormal head postures and nonmotor symptoms such as anxiety. Although structural and functional brain alterations have been reported, findings remain inconsistent, and the neurobiological mechanisms underlying motor and nonmotor symptoms remain incompletely understood.

**Methods:**

Thirty-five ICD patients and twenty-eight matched healthy controls underwent structural MRI and resting-state fMRI. Voxel-based morphometry was used to assess gray matter volume (GMV) differences. Seed-based resting-state functional connectivity (rsFC) analyses were performed using regions with significant structural alterations. Partial correlation and mediation analyses examined associations among brain measures, motor severity, and mood symptoms.

**Results:**

ICD patients showed reduced GMV in the left paracentral lobule (PCL) and right middle temporal gyrus (MTG). The left PCL exhibited altered connectivity with prefrontal, temporal, and thalamic regions, indicating disruption of cerebello-thalamo-cortical pathways. The right MTG showed decreased connectivity with the left temporal pole and increased connectivity with the right middle frontal gyrus, suggesting compensatory mechanisms for cognitive processing. GMV reduction in the left PCL significantly mediated the relationship between ICD status and anxiety symptoms.

**Discussion:**

These findings support ICD as a network disorder involving both motor and cognitive-affective circuits. Structural alterations in the PCL and MTG and their connectivity patterns may underlie motor dysfunction and nonmotor symptoms such as anxiety. Multimodal neuroimaging biomarkers may help guide targeted therapeutic interventions and improve clinical outcomes in ICD.

## Introduction

Dystonia is a heterogeneous group of movement disorders characterized by sustained or intermittent muscle contractions, causing abnormal, often repetitive movements, postures, or both (1). Among these, isolated cervical dystonia (ICD), also known as spasmodic torticollis, is by far the most common form of isolated dystonia, accounting for approximately 40% of all cases (2, 3). ICD typically manifests in adulthood and is characterized by involuntary muscle contractions of the neck and shoulders, leading to abnormal head postures, tremor, and pain (2). The likelihood of a current or lifetime diagnosis of a psychiatric illness of any type in ICD is as high as 91.4% compared to 35% of adults in the general population (4). These nonmotor manifestations often exert a greater impact on health-related quality of life than motor symptoms themselves (5–7).

Recently, neuroimaging studies employing structural MRI (sMRI), and functional MRI (fMRI) have advanced our understanding of the pathophysiology of dystonia (8), revealing alterations in brain regions implicated in sensorimotor integration, motor control, and affective processing, such as the basal ganglia, paracentral lobule, cerebellum, prefrontal and temporal cortex (9). However, different studies have reported varying findings regarding gray matter volume (GMV) alterations in ICD. Some studies observed both increases and decreases in GMV across different brain regions (10, 11), such as increased GMV in the thalamus, caudate head, superior temporal lobe, and cerebellum, while decreased in the putamen bilaterally (11). One study reported only increased GMV in the bilaterally globus pallidus internus, nucleus accumbens, prefrontal cortex and the left inferior parietal lobe (12). Other studies found exclusively gray matter atrophy of thalamus (13, 14), cerebellum and primary motor cortex (15, 16), prefrontal cortex (17) in patients with ICD compared to healthy controls, while some study did not detect any regional structural differences between patients with ICD and healthy controls (18).

Meanwhile, studies using fMRI method also have revealed widespread functional alterations in ICD, including decreased activity and connectivity in the primary somatosensory cortex and premotor areas, alongside increased cerebellar activation correlated with symptom severity (18). Additionally, disrupted functional connectivity between the cerebellum and basal ganglia networks, as well as hypoactivation in posterior cerebellar lobules during motor timing tasks, have been reported in ICD patients (19). A recent study voxel-wise global-brain functional connectivity (GFC) analysis revealed significantly reduced GFC in the right precentral gyrus and right supplementary motor area (SMA) in patients with ICD (20). Notably, GFC in the right precentral gyrus was negatively correlated with symptom severity. These findings highlight abnormal cerebello-thalamo-cortical and basal ganglia circuits as key features underlying ICD pathophysiology (21). However, the results across studies remain heterogeneous, potentially due to differences in sample characteristics, clinical subtypes. Some studies have focused solely on GMV alterations, while others have explored functional connectivity changes without considering underlying structural deficits. Consequently, integrative analyses combining structural and functional imaging remain limited, hindering a comprehensive understanding of the neurobiological underpinnings of ICD.

To address these gaps, the present study employed a multimodal MRI approach to investigate GMV alterations and associated resting-state functional connectivity (rsFC) changes in patients with ICD compared to healthy controls. Using voxel-based morphometry, we identified regions with significant GMV differences and then assessed whether these structural abnormalities were accompanied by altered rsFC patterns. We further explored their relationships with motor severity, quality of life, depression, and anxiety symptoms. Additionally, mediation analyses were performed to examine whether GMV alterations mediated the association between disease status and mood symptoms with structural equation modeling. By integrating structural and functional imaging with mediation analyses, this study aims to provide novel insights into the neural substrates underlying both motor and nonmotor symptoms in ICD, informing potential therapeutic targets.

## Methods

### Participants

Participants were recruited from the outpatient clinic for movement disorders and were diagnosed with adult-onset ICD following the criteria (3, 22) by two senior neurologists. Patients who matched the following criteria were included in the study: (1) isolated cervical dystonia; (2) no history of botulinum toxin treatment, related drug therapy, or operation in the previous 3 months; (3) no history of serious physical or neuropsychiatric disorders; and (4) right-handedness. The patients were excluded according the following criteria: (1) age <18 years; (2) genetic findings associated with the onset of dystonia, and (3) had medical implants contraindicated for undergoing MRI. Finally, thirty-five patients diagnosed with ICD and twenty-eight age and sex matched healthy controls were recruited. All participants completed safety questionnaires for MRI and provided written informed consent in accordance with the Declaration of Helsinki. This study was approved by the Ethics Committee of First Teaching Hospital of Tianjin University of Traditional Chinese Medicine.

### Clinical assessments

Motor symptom severity in ICD patients was assessed using the motor subscore of the Toronto western spasmodic torticollis rating scale (TWSTRS) (23). Disease-specific quality of life was evaluated using the Craniocervical dystonia questionnaire-24 (CDQ-24) (24), which is specifically designed for patients with craniocervical dystonia and is sensitive to both motor and psychosocial burden. To assess mood symptoms, we used the Hamilton depression rating scale (HAMD) (25), and the self-rating anxiety scale (SAS) (26). Importantly, the HAMD and SAS assessments were administered to both ICD patients and healthy controls, enabling group-level comparisons of depressive and anxiety symptoms, and allowing us to explore the nonmotor symptom burden characteristic of dystonia.

### MRI acquisition

All participants underwent high-resolution T1-weighted structural MRI and resting-state fMRI scans on a 3 T GE Discovery MR750 scanner. All subjects underwent scans with foam padding and earplugs to minimize head movement and reduce scanner noise. A high-resolution T1-weighted brain volume (BRAVO) 3D MRI sequence with 128 contiguous sagittal slices was performed with the scan parameters of repetition time (TR) = 8.2 ms; echo time (TE) = 3.2 ms; inversion time = 450 ms; field of view (FOV) = 256 × 256 mm^2^; slice thickness = 1.0 mm; no gap; flip angle (FA) = 12°; matrix = 256 × 256; and voxel size = 1 mm^3^. The resting-state fMRI scans were performed by an echo planar imaging (EPI) sequence with the scan parameters of TR = 2000 ms, TE = 30 ms, FA = 90°, matrix = 64 × 64, FOV = 240 × 240 mm^2^, slice thickness = 3 mm, gap = 1 mm. Each brain volume comprised 36 axial slices, and each functional run contained 188 volumes. During the fMRI scans, all of the subjects were instructed to keep their eyes closed, to relax and to move as little as possible.

### Data preprocessing

VBM preprocessing was performed using the CAT12 toolbox with the accompanying methodology: bias correction, segmentation, the creation of population-specific tissue templates, spatial normalization using the DARTEL technique, and smoothing with an 8 mm × 8 mm × 8 mm full-width. After these preprocessing steps, we acquired the normalized, modulated, and smoothed GMV images, and each voxel represented volume information. Total intracranial volume (TIV) was computed for each participant.

Resting-state fMRI data were preprocessed automatically using the DPABI software (27). The first 10 volumes were discarded to allow for signal equilibrium. Preprocessing steps included slice-timing correction, realignment for head motion correction, and exclusion of participants with head movements exceeding 2 mm in translation or 2° in rotation. Images were then spatially normalized to the MNI EPI template via SPM12 and resampled to 3 × 3 × 3 mm^3^ voxels, followed by smoothing with an 8 mm FWHM Gaussian kernel. The resulting time series underwent linear detrending and band-pass filtering (0.01–0.08 Hz). Nuisance covariates, including Friston-24 head motion parameters, white matter signal, cerebrospinal fluid signal, and global signal, were regressed out. Framewise displacement (FD) value was calculated for each participant (28), and scrubbing was performed by removing time points with FD > 0.2 mm to minimize motion-related artifacts.

### VBM analysis

We conducted the voxel-based comparisons to distinguish the brain regions that showed group differences in GMV by utilizing the two-sample *t*-test, with age, gender, FD, TIV, age-onset and disease duration as covariates. The assessment of whether this group difference is significant was performed using a non-parametric permutation-based two-sample *t*-test (with 5,000 permutations) and further employing threshold-free cluster enhancement (TFCE) combined with false discovery rate (FDR) correction at *p* < 0.05 to control for false positives due to multiple comparisons at the voxel level.

### Resting-state functional connectivity analysis

Seed-based functional connectivity analysis was conducted using the regions showing significant GMV differences as region of interests (ROIs). Thus, ROI-based FC analysis was performed to calculate the FC maps of each ROI using the DPABI software. For each subject, the correlation coefficients between the mean time series of each ROI and that of each voxel of the whole brain were computed and converted to *z*-values, using Fisher’s *r*-to-*z* transformation to improve the normality. Similar to the VBM analysis, the two-sample *t*-test analysis was performed to test the group difference between the rsFCs of each of the ROIs, age, gender, FD, TIV, age-onset and disease duration were entered as covariates. The correction for multiple comparisons was performed using FDR correction at *p* < 0.05, cluster size >30.

### Statistical analysis

In addition to neuroimaging analyses, we conducted supplementary statistical analyses in R v4.4.3^1^ to investigate clinical variable relationships and group-level differences. Group comparisons of age, HAMD, and SAS between ICD patients and healthy controls were performed using two-sample *t*-tests, while group differences in sex were evaluated using chi-square (*χ^2^*) tests. These analyses were conducted using the *stats* package. Statistical significance was defined as two-tailed *p* < 0.05.

We computed Pearson correlation coefficients among clinical measures, including TWSTRS motor score, CDQ-24, HAMD, and SAS, to explore the associations between motor symptoms, disease-related quality of life, and mood disturbances. Correlation matrices were generated using the *psych* package, and significance was set at two-tailed *p* < 0.05.

To examine brain behavior associations, we extracted mean GMV from the significant GMV differences identified in the VBM analysis. These regional GMV values of ICD patients were then correlated with clinical scores (TWSTRS, CDQ-24, HAMD, SAS) using partial Pearson correlation (controlling for age, sex, FD, TIV, age-onset and disease duration) via the *ppcor* package in R.

### Mediation analysis

To further explore the potential mediating role of significant GMV differences regions in the relationship between disease status and mood symptoms, we performed mediation analyses using structural equation modeling. Specifically, we tested whether GMV in regions showing significant group differences mediated the association between group status (ICD patients vs. healthy controls; coded as a binary variable) and mood symptom severity, as assessed by HAMD and SAS scores.

The mediation models were constructed with group status as the independent variable (X), regional GMV as the mediator (M), and mood symptoms (HAMD or SAS) as the dependent variable (Y). Age and gender were included as covariates in all models. Standardized estimates were calculated using the *lavaan* package in R v4.4.3. The significance of indirect effects was assessed via bootstrapping procedures (5,000 resamples), and *p*-values <0.05 were considered statistically significant.

## Results

### Demographic and clinical characteristics

A total of 63 participants were included in the analysis, comprising 35 patients with ICD and 28 healthy controls. The demographic and clinical characteristics of the participants are summarized in Table 1. There was no significant difference in age, gender and HAMD scores between the ICD group and the healthy controls group. However, the ICD group showed significantly higher anxiety levels compared to healthy controls, as measured by the SAS (*p* < 0.001). As TWSTRS and CDQ-24 are disease-specific scales, they were only administered in the ICD group, yielding mean scores of 30.90 ± 13.69 and 53.66 ± 18.57, respectively.

**Table 1:** The demographic and clinical characteristics of the participants.

Variables	HC group	ICD group	Statistic value	*p* value
Gender	17 M/11F	14M/21F	1.91	0.167
Age	38.61 ± 8.41	34.74 ± 11.06	1.57	0.121
TWSTRS	NA	30.90 ± 13.69	NA	NA
CDQ-24	NA	53.66 ± 18.57	NA	NA
HAMD	6.31 ± 3.67	6.57 ± 2.70	0.32	0.750
SAS	24.61 ± 2.06	29.49 ± 5.91	−4.55	**<0.001**
FD	0.127 ± 0.022	0.117 ± 0.018	1.84	0.07
Age-onset	NA	33.61 ± 10.23	NA	NA
Disease duration	NA	15.62 ± 23.27	NA	NA

CDQ-24, Craniocervical dystonia questionnaire-24; FD, Framewise displacement; HAMD, Hamilton depression rating scale; ICD, isolated cervical dystonia; NA, not applicable; SAS, self-rating anxiety scale; TWSTRS, Toronto western spasmodic torticollis rating scale (TWSTRS). Bold values indicate statistically significant differences (*p* < 0.05).

### VBM alterations of ICD patients

**Figure 1:**
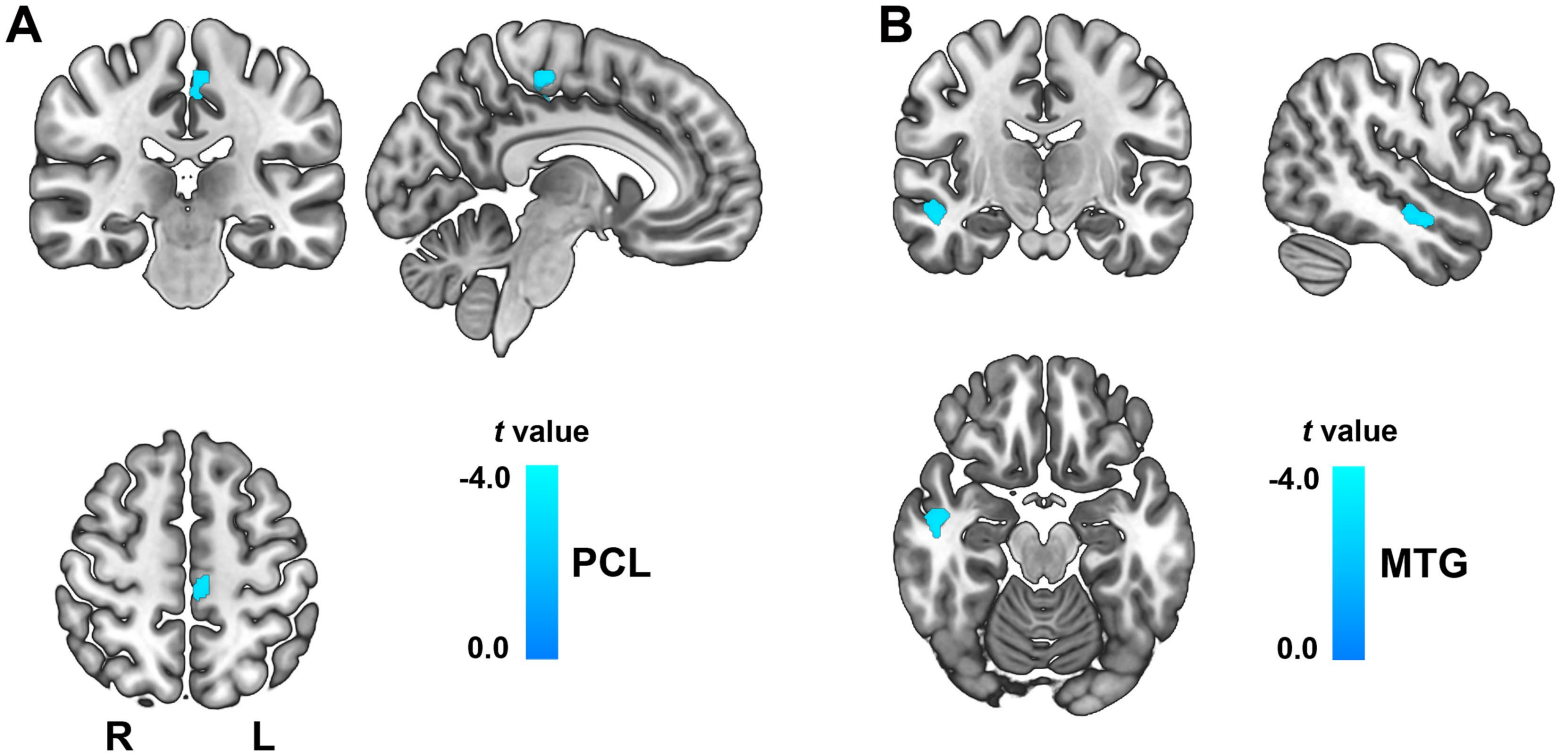
GMV changes between ICD patients and healthy controls. The color bar represents *t*-values. There were significantly decreased GMV in the left PCL **(A)** and right MTG **(B)**. GMV, gray matter volume; ICD, isolated cervical dystonia; L, left; MTG, middle temporal gyrus; PCL, paracentral lobule.

Compared to healthy controls, ICD patients exhibited significantly decreased GMV in the left paracentral lobule (PCL, peak MNI coordinates: x = −9.3, y = −22.1, z = 54; cluster size = 196 voxels; *t* = −3.33; Cohen’s *d* = −0.84, Figure 1A), and right middle temporal gyrus (MTG, peak MNI coordinates: x = 49.1, y = −14.2, z = −18; cluster size = 227 voxels; *t* = −3.29; Cohen’s *d* = −0.83, Figure 1B). These differences remained significant after TFCE and FDR correction (*p* < 0.05). In addition, there were no regions demonstrating a significant increased GMV in ICD patients.

### Functional connectivity alterations of ICD patients

Seed-based rsFC analysis was conducted using the left PCL and the right MTG as ROIs with significantly reduced GMV in ICD patients. Group-level comparisons revealed both decreased and increased functional connectivity patterns in ICD patients compared to healthy controls. All results were significant after FDR correction at *p* < 0.05, cluster size > 30.

**Figure 2:**
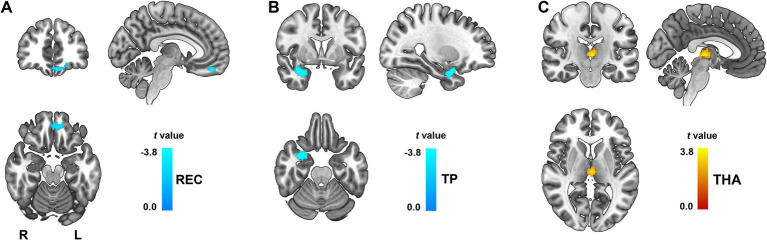
Altered functional connectivity of the left PCL in ICD patients. Functional connectivity differences were observed using the left PCL as the seed region. **(A)** Decreased rsFC between left PCL and the left REC **(A)** and right TP **(B)**. Increased rsFC between left PCL and the bilateral THA **(C)**. All results were FDR corrected at *p* < 0.05. L, left; PCL, paracentral lobule; R, right; REC, rectus gyrus; THA, thalamus; TP, temporal pole.

Compared to the healthy controls, decreased rsFC was observed between left PCL and the left rectus (REC, peak MNI coordinates: x = −7, y = 49, z = −20; cluster size = 38 voxels; *t* = −3.46; Cohen’s *d* = −0.88, Figure 2A), right temporal pole (TP, peak MNI coordinates: x = 31, y = 5, z = −23; cluster size = 67 voxels; *t* = −3.31; Cohen’s *d* = −0.84, Figure 2B), increased rsFC was identified between left PCL and the bilateral thalamus (THA, peak MNI coordinates: x = −4, y = −21, z = 3; cluster size = 36 voxels; *t* = 3.00; Cohen’s *d* = 0.76, Figure 2C) in the ICD patients.

**Figure 3:**
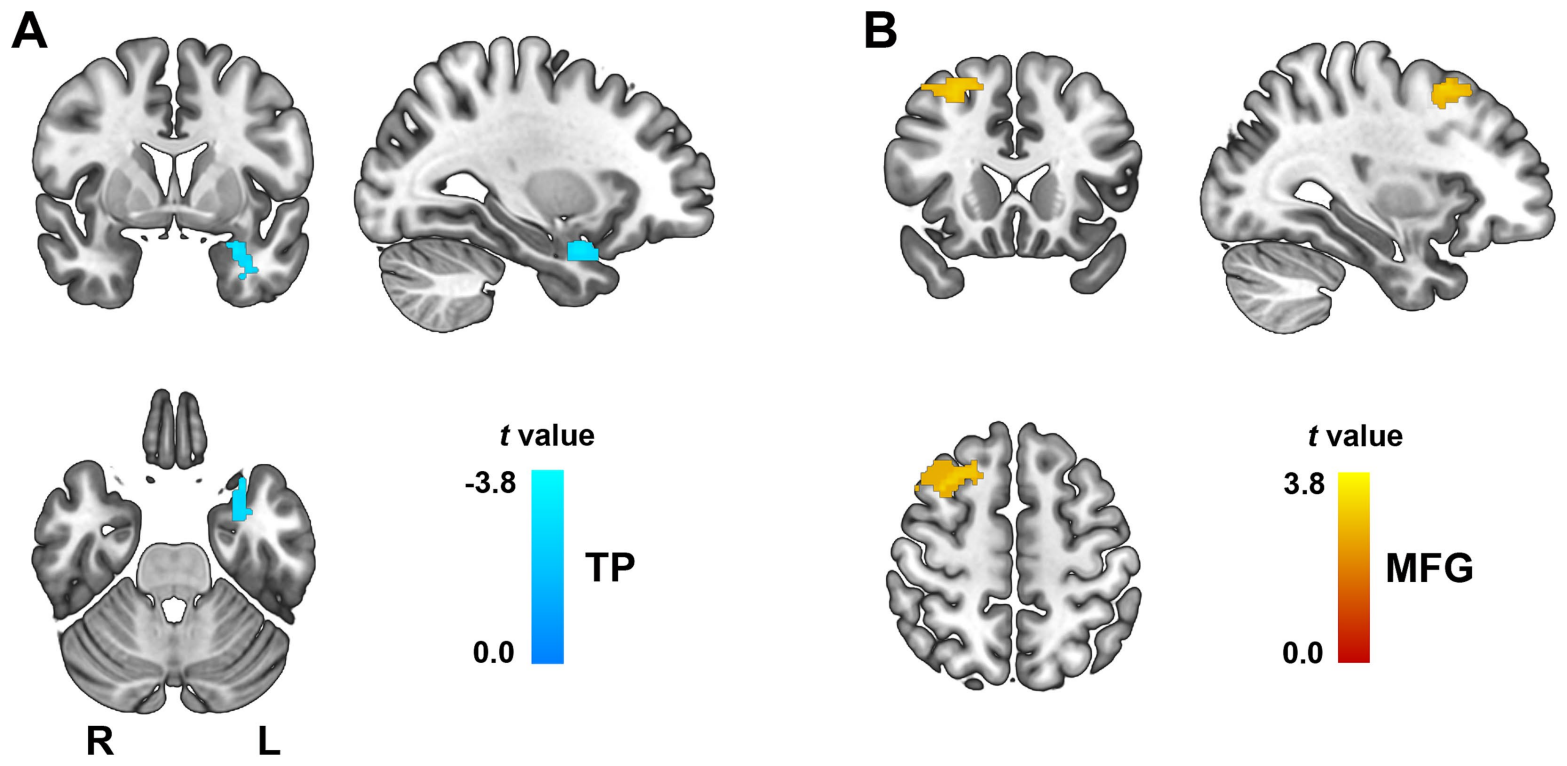
Altered functional connectivity of the right MTG in ICD patients. Functional connectivity differences were observed using the right MTG as the seed region. **(A)** Decreased rsFC between right MTG and the left TP. **(B)** Increased rsFC between left MTG and the right MFG. All clusters survived FDR correction at *p* < 0.05. L, left; MFG, middle frontal gyrus; MTG, middle temporal gyrus; TP, temporal pole.

Similarly, we found decreased rsFC between right MTG and the left TP (peak MNI coordinates: x = −30, y = 4, z = −24; cluster size = 47 voxels; *t* = −2.73; Cohen’s *d* = −0.69, Figure 3A), increased rsFC between left MTG and the right middle frontal gyrus (MFG, peak MNI coordinates: x = 35, y = 12, z = 45; cluster size = 145 voxels; *t* = 2.52; Cohen’s *d* = 0.64, Figure 3B) in ICD patients. These findings suggest disrupted connectivity between temporal/frontal regions and key affective and executive hubs, with both hypo- and hyper-connectivity potentially reflecting maladaptive reorganization in response to underlying structural deficits.

### Correlations among clinical measures and GMV alterations

**Figure 4:**
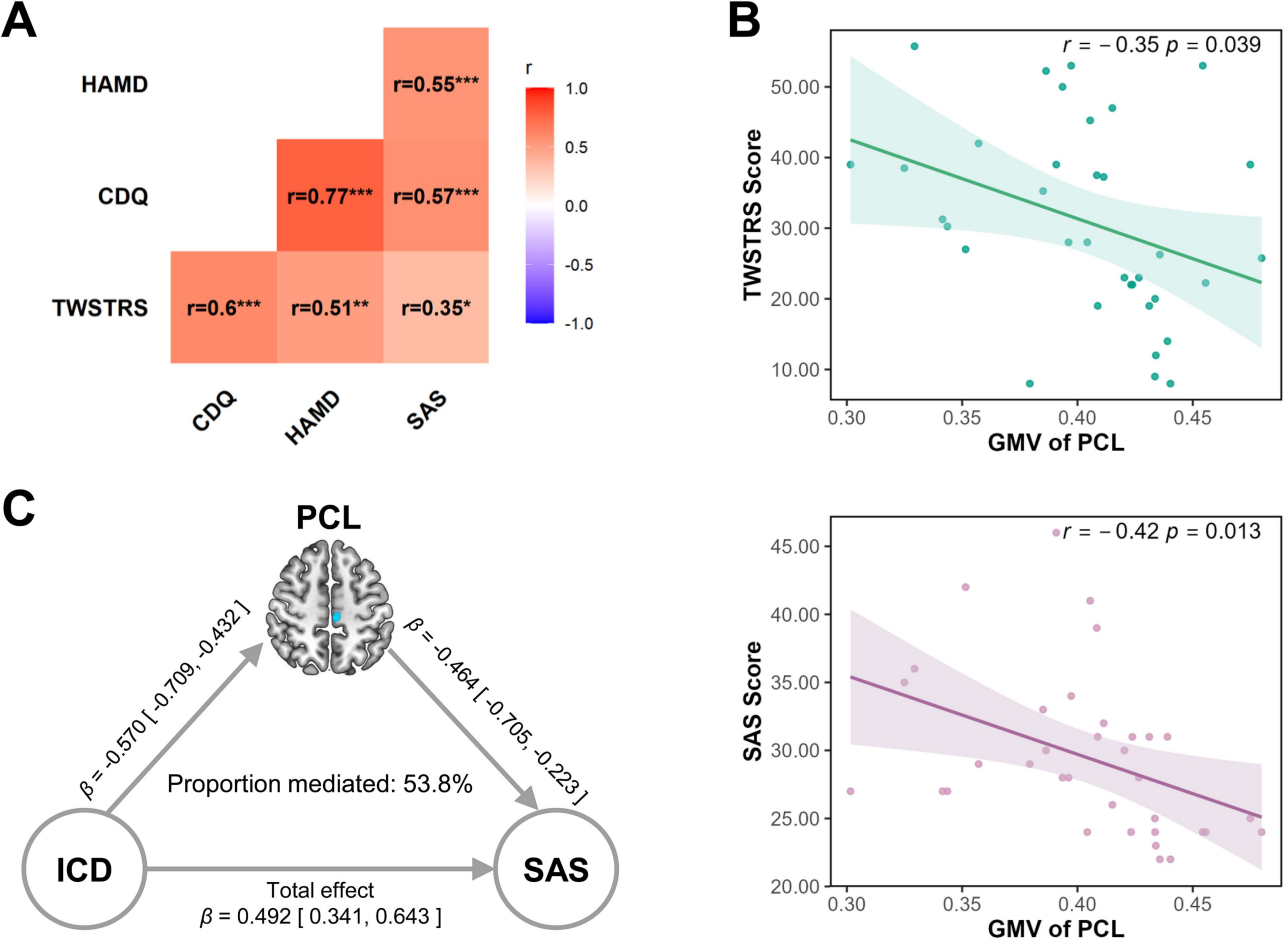
Clinical correlations and mediation effect of left PCL in ICD patients. Combined correlation analysis and mediation model in patients with isolated cervical dystonia (ICD). **(A)** Heatmap showing Pearson correlation coefficients (*r* values) among clinical measures in ICD patients, including motor severity (TWSTRS), disease-specific quality of life (CDQ-24), depression severity (HAMD), and anxiety levels (SAS). Stronger correlations are indicated by deeper red hues, while weaker or non-significant correlations appear in lighter shades. Statistical significance is denoted as follows: * *p* < 0.05, ** *p* < 0.01, *** *p* < 0.001. **(B)** Scatter plots showing that GMV in the left PCL is associated with higher TWSTRS score, while GMV in the left PCL is negatively correlated with SAS scores. All correlations were adjusted for age and sex, with shaded regions representing 95% confidence intervals. **(C)** Mediation path diagram illustrating that reduced GMV in the left PCL partially mediates the association between disease status (ICD vs. controls) and anxiety severity (SAS). Standardized path coefficients (*β*) with 95% confidence intervals are presented along each arrow. CDQ-24, Craniocervical dystonia questionnaire-24; GMV, gray matter volume; HAMD, Hamilton depression rating scale; ICD, isolated cervical dystonia; PCL, paracentral lobule; SAS, self-rating anxiety scale; TWSTRS, Toronto western spasmodic torticollis rating scale.

We conducted correlation analyses to investigate the relationships among clinical variables and their associations with regional GMV alterations in patients with ICD. As shown in Figure 4A, the TWSTRS motor score was significantly positively correlated with CDQ-24 (*r* = 0.60, *p* < 0.001), HAMD (*r* = 0.51, *p* < 0.01), and SAS (*r* = 0.35, *p* < 0.05), indicating that greater motor severity was associated with poorer quality of life and higher levels of depressive and anxiety symptoms. The CDQ-24 score showed robust positive correlations with both HAMD (*r* = 0.77, *p* < 0.001) and SAS (*r* = 0.57, *p* < 0.001), highlighting the strong contribution of mood disturbances to the perceived disease burden in ICD. Furthermore, HAMD and SAS scores were also correlated with each other (*r* = 0.55, *p* < 0.001). In terms of brain-behavior relationships, partial correlation analyses controlling for age and sex revealed that GMV in the left PCL was negatively correlated with TWSTRS (*r* = −0.35, *p* = 0.039, Figure 4B) and SAS (*r* = −0.42, *p* = 0.013) (Figure 4B). These findings underscore the close interplay between structural brain changes and clinical manifestations in ICD, and reinforce the role of affective symptoms in shaping disease burden.

### Mediation analysis

We examined whether GMV in the left PCL or right MTG mediated the association between group status and mood symptoms (HAMD and SAS). Among the four tested models, only the pathway involving GMV in the left PCL and anxiety (SAS) demonstrated a significant indirect effect (Figure 4C). Specifically, patients with ICD showed significantly reduced GMV in the left PCL compared to controls (path a: standardized *β* = −0.570, *p* < 0.001, 95% CI [−0.709, −0.432]). In turn, lower PCL GMV was associated with higher anxiety scores (path b: standardized *β* = −0.464, *p* < 0.001, 95% CI [−0.705, −0.223]). The indirect effect (ab) was significant (standardized *β* = 0.265, *p* < 0.001, 95% CI [0.125, 0.405]), yielding a proportion mediated of approximately 53.8%. These findings indicate that GMV alterations in the left PCL statistically account for a substantial portion of the group difference in anxiety symptoms. No significant statistical mediation effects were observed in the models involving HAMD scores or MTG/PCL and SAS scores or MTG (all *p* > 0.05).

## Discussion

In this study, we investigated GMV alterations and associated rsFC changes in patients with ICD. Our findings revealed significant GMV reductions in the left PCL and right MTG in ICD patients. Furthermore, these structural abnormalities were accompanied by both decreased and increased rsFC between affected regions and key cortical and subcortical areas. Importantly, mediation analysis demonstrated that the GMV reduction in the left PCL partially mediated the relationship between ICD status and anxiety symptoms, suggesting a potential neural mechanism linking structural alterations to nonmotor symptomatology.

The PCL, which largely encompasses the primary motor cortex, takes part in the motor planning, control and execution of movement (29). Our finding of reduced GMV in the medial aspect of the left PCL, extends previous neuroimaging studies in ICD that have reported cortical thickness and GMV reductions in primary motor cortex (15, 16, 30, 31). Although no separate significant clusters were observed in the lateral M1 proper in our voxelwise analysis, the medial PCL peak likely reflects partial involvement of M1, particularly its medial somatotopic representation. Thus, our result complements previous M1 findings and may reflect a common pathophysiological substrate manifesting in slightly different spatial locations across studies. The GMV reduction may reflect a loss of neurons or a reduction in synaptic density, or both. Such microstructural alterations would be consistent with neurophysiological evidence demonstrating impaired intracortical inhibition (32–34) as well as cerebellar-brain inhibition deficits in dystonia (35–37). We found that the left PCL exhibited altered functional connectivity with both cortical and subcortical regions. Specifically, reduced connectivity with the left rostral entorhinal cortex and right temporal pole may reflect disrupted integration between sensorimotor and higher-order cognitive-emotional networks. Such dysconnectivity is consistent with the broader network-based pathophysiology of dystonia and may stem from impaired intracortical inhibitory processes within the sensorimotor cortex (32). In contrast, increased connectivity between the PCL and thalamus may reflect upregulation of thalamocortical relay activity in response to reduced cortical inhibition, mechanisms that have been proposed in prior neurophysiological models of dystonia (35). This interpretation aligns with previous evidence of cerebello-thalamo-cortical (CTC) circuit dysfunction and white matter abnormalities in both manifesting and non-manifesting dystonia gene carriers (38–41). These structural connectivity alterations likely compromise sensorimotor integration and motor control, core features disrupted in dystonia. Interestingly, while altered cortico-cortical connectivity from the sensory cortex to primary motor cortex has been implicated in motor control in healthy individuals and other movement disorders, it remains underexplored in ICD (42). Future studies integrating DTI-based structural connectivity with fMRI-derived functional connectivity may clarify the contribution of these pathways to the observed structural abnormalities.

Additionally, we found significantly reduced GMV in the right MTG in patients with ICD, consistent with previous studies (31, 43). The MTG is a key associative region implicated in multimodal integration and cognitive processing (44). It is anatomically connected to visual and auditory networks as well as frontoparietal control systems, and plays an important role in attentional modulation, object recognition, and spatial cognition (45, 46). While not directly involved in fine motor execution, structural alterations in the MTG may contribute to broader impairments in attentional reorientation, or cognitive-emotional regulation. Given that patients with ICD often exhibit cognitive impairments (44), including deficits in executive function and visuospatial processing, the observed MTG atrophy may underlie aspects of the nonmotor symptomatology. Moreover, our seed-based rsFC analyses revealed decreased functional connectivity between the right MTG and the left TP, alongside increased connectivity with the right MFG. The enhanced connectivity with prefrontal regions might reflect compensatory recruitment of executive resources to maintain cognitive performance, whereas reduced connectivity with the temporal pole could signify disrupted integration within temporal lobe structures critical for semantic memory and social cognition (45). Further research combining structural and task-based functional imaging is needed to clarify the specific cognitive domains affected and their relationship with MTG structural and functional abnormalities in ICD.

Correlation analyses revealed significant relationships between GMV reductions and clinical measures. Notably, reduced GMV in the left PCL correlated with higher motor severity and anxiety levels. These findings support previous studies showing that structural brain alterations are not only linked to motor symptoms but also to nonmotor manifestations such as depression and anxiety (4). Our mediation analysis provides novel insight into the potential mechanistic link between structural brain changes and mood symptoms in ICD. Previous study using DTI have found that individuals with higher trait anxiety have stronger connections between the PCL and the amygdala (47). The finding that reduced GMV in the left PCL partially and statistically mediated the relationship between ICD and anxiety symptoms suggests that sensorimotor cortical alterations may influence emotional processing and regulation.

Several limitations should be acknowledged. First, the cross-sectional design limits causal inferences about the relationship between brain changes and clinical symptoms. Although we performed mediation analyses to explore statistical pathways linking group status, brain structure, and anxiety symptoms, these results do not imply directional or causal relationships. Longitudinal studies are needed to determine whether structural and functional changes precede symptom onset or emerge as a consequence. Second, although our sample size is comparable to prior neuroimaging studies in dystonia, may still limit statistical power particularly for detecting subtle effects in expected regions such as the lateral primary motor cortex and cerebellum, or for testing complex models such as mediation. Third, our seed selection strategy was based on regions showing significant structural alterations, which may have excluded functionally abnormal but structurally preserved regions. Whole-brain or data-driven approaches may provide a more comprehensive view of network dysfunction. Finally, detailed clinical laterality data (e.g., side of onset or head deviation) were not systematically collected, limiting our ability to relate hemispheric findings to symptom asymmetry.

In conclusion, our multimodal neuroimaging study reinforces the concept of ICD as a disorder of distributed brain networks rather than isolated anatomical loci. The involvement of the PCL, MTG and their associated circuits provides a neurobiological framework for understanding both the motor and nonmotor manifestations of the disease. Such insights are critical for advancing toward personalized treatment strategies that address the full spectrum of ICD pathology.

## Data Availability

The raw data supporting the conclusions of this article will be made available by the authors, without undue reservation.

## Footnotes

1
https://www.Rproject.org/
